# Occurrence of human e nterovirus in tropical fish and shellfish and their relationship with fecal indicator bacteria

**DOI:** 10.14202/vetworld.2018.1285-1290

**Published:** 2018-09-18

**Authors:** Manjusha Lekshmi, Oishi Das, Sanath Kumar, Binaya Bhusan Nayak

**Affiliations:** Department of Post-Harvest Technology, Quality Control Laboratory, ICAR-Central Institute of Fisheries Education, Versova, Mumbai, Maharashtra, India

**Keywords:** coliforms, enterovirus, fish, indicator bacteria, reverse transcription-polymerase chain reaction, shellfish

## Abstract

**Aim:**

Human enteroviruses in fish and shellfish are a health concern worldwide. Human infections occur due to the consumption of raw or insufficiently cooked fish or shellfish. The objective of this study was to determine the occurrence of human enteric viruses belonging to Enterovirus (EV) group in seafood in Mumbai and to correlate their occurrence with the bacterial indicators of fecal contamination.

**Materials and Methods:**

Samples of fresh fish and shellfish collected from fish landing centers and retail fish markets were analyzed by virus concentration, nucleic acid extraction, and reverse transcription-polymerase chain reaction (RT-PCR). Bacterial indicators of fecal contamination were estimated by the most probable number technique. The relationship between the presence of virus and fecal indicators was determined by statistical analysis.

**Results:**

A total of 89 samples comprising of fish, shrimps, oysters, clams, and mussels were screened in this study. EV was detected in 32 (35.95%) samples, and all the virus-positive samples belonged to bivalve molluscan group. None of the finfish and crustacean shellfish samples was positive for the enteric viruses. Clams were found to be the most contaminated with 48.4% of the samples being positive for EV. The prevalence of enteric viruses in seafood samples showed a strong positive correlation with the bacteriological indicators of fecal contamination, suggesting that fecal coliform bacteria are good indicators of EVs in tropical seafood.

**Conclusion:**

The presence of EVs in seafood is a public health hazard. Increasing level of coastal water contamination from anthropogenic sources is the primary reason for the contamination of seafood with EVs. Continuous monitoring of coastal waters and seafood for enteric viruses will help to ensure the safety of fish and shellfish for human consumption.

## Introduction

Human enteric viruses are pathogens of public health significance responsible for food- and water-borne gastrointestinal diseases worldwide [1]. Infections are acquired through fecal route, with sewage and urban wastewater run-off being the major sources of contamination [2,3]. Enteric viruses are shed in extremely high numbers in the feces of infected individuals, typically between 10^5^ and 10^11^ virus particles per gram of stool [4]. Polioviruses (PVs) and enteroviruses (EVs) are among the most common genera of the Picornaviridae family of enteric viruses that infect humans [5]. They are small, non-enveloped RNA viruses with a capsid of about 30-nm diameter and icosahedral symmetry [6]. The EV genome is comprised of a single-stranded polyadenylated RNA of a positive sense of about 7500 bases with a single open reading frame (ORF) encoding a polyprotein with 2200 amino acids. The polyprotein ORF is flanked by a long untranslated region at the 5′ end (5′UTR) and a short UTR at the 3′ end [7]. A 22 amino acid virus-encoded protein is covalently linked to the 5′ end. These viruses are highly resistant to hostile environmental conditions such as high temperature, pH, and radiation which facilitate their survival for long in the environment. Their small size, genome type, and non-enveloped capsid structure also play important roles in their survival [8].

Seafood such as shellfish are important vehicles of human enteric viruses [5]. Filter-feeding shellfish such as clams and oysters filter large volumes of water as part of their feeding activities and thus accumulate bacterial and viral pathogens [9]. Crustaceans such as crabs and shrimps acquire enteric viruses when they feed on contaminated oysters or other organisms and act as sources of transmission to humans [10]. Consumption of raw or partially cooked contaminated shellfish can lead to the transmission of disease. Many outbreaks have been reported due to the consumption of contaminated shellfish worldwide [11]. In general, enteric viral infections are mild and self-limiting. However, infections can occasionally lead to complications involving hepatitis, conjunctivitis, allergies, encephalitis, myocarditis, pericarditis, and foot and mouth disease [12,13].

Despite their human health importance, studies on the prevalence of EVs in seafood from India are sparse [9,14]. Therefore, the present study was undertaken to investigate the prevalence of EVs in seafood samples from Mumbai and the relationship between their occurrence and bacteriological indicators of fecal contamination.

## Materials and Methods

### Ethical approval

Ethical approval was not required in this study since no live animals were used in the experiments.

### Sample collection and processing

A total of 89 samples of fish and shellfish collected from fish landing centers, retail markets, and supermarkets were analyzed during the period from January 2016 to September 2017. The samples were collected in sterile plastic bags, transported to the laboratory in insulated ice boxes, and processed immediately. Live bivalve samples of clams, oysters and mussels were opened using a sterile shucking knife and the digestive glands were collected along with the intravalvular fluid. In the case of shrimps, hepatopancreas was dissected and collected, while muscle and skin from finfish samples were used for further processing.

### Concentration of virus

The samples were subjected to the concentration process for viruses [15] before the extraction of viral nucleic acids. Briefly, samples of digestive glands, hepatopancreas, muscle, and skin were homogenized in a Stomacher (Seward Stomacher 80, Lab system, London, UK). The homogenate (50 g) was mixed with equal volume of glycine buffer (0.5 M glycine, 0.15 M NaCl, pH 9.5), followed by magnetic agitation for 15 min at room temperature to release viruses from the tissue. The homogenate was clarified by centrifugation at 10,000 ×g for 10 min at 4°C. The supernatant was recovered, and an equal volume of 3% meat extract was added to precipitate and adsorb the viruses. After adjusting the pH to 3.5, the supernatant was agitated for 30 min at room temperature and centrifuged at 10,000× *g* for 15 min at 4°C. The pellet was resuspended in 5 mL of phosphate-buffered saline (PBS) (pH 7). The extract was precipitated with 50% PEG 6000 and added in a ratio of 1:4 (v/v). The pH was adjusted to 7.2, and the solution was incubated overnight at 4°C. The precipitate was clarified by centrifugation at 10,000× *g* at 4°C for 45 min. The pellets were resuspended in 5 mL PBS (pH 7), aliquoted, and stored at −20°C.

### RNA extraction and cDNA synthesis from viral concentrate

Total RNA was extracted from the concentrated samples using the SV Total RNA Isolation Kit (Promega, USA) according to the manufacturer’s protocol. The extracted RNA was converted into cDNA using GoScript Reverse Transcription System (Promega, USA). Polymerase chain reaction (PCR) was performed on the cDNA template using virus group-specific primers.

### Detection of EV by PCR

For the detection of EV group, nested PCR primers ENV-F (CAAGCACTTCTGTTTCCCCGG), ENV-R1 (ATTGTCACCATAAGCAGCCA) and ENV-R2 (CTTGCGCGTTACGAC) were used [16]. cDNA (3 µL) was subjected to PCR amplification in a 30 µL volume comprising of 15 µL of EmeraldAmp PCR 2X Master Mix (TaKaRa, Japan) and 30 picomoles of forward and reverse primers. In the case of nested PCR, 3 µL of the first step PCR product was used as the template DNA for the second round of amplification. All amplifications were done in a SimpliAmp Thermal Cycler (Thermo Fisher Scientific, USA). The PCR products were analyzed by electrophoresis on 2.0% agarose gels, stained with ethidium bromide (0.5 µg/mL), and photographed using a gel documentation system (Bio-Rad, USA). A 435 bp cloned fragment of EV was used as the positive control in PCR assays.

### Determination of fecal coliform count by most probable number (MPN) technique

Fecal coliform and *Escherichia coli* counts were determined in all the samples of fish and shellfish following conventional method [17]. Briefly, a 25 g of the seafood sample was homogenized in 225 mL of Butterfield’s phosphate-buffered water for 2 min. Appropriate 10-fold dilutions of the homogenate were prepared and inoculated into lauryl sulfate tryptose broth tubes (3-tube MPN) and incubated at 37°C for 24-48 h. Two loopfuls from positive tubes, indicated by turbidity and gas production, were inoculated into *E. coli* broth and incubated at 44.5°C for 24-48 h. *E. coli* was isolated from positive tubes on eosin methylene blue agar plates and typical colonies of *E. coli* were identified by indole, methyl red, Voges–Proskauer and citrate tests.

### Statistical analysis

Association between EV-positive samples and the bacteriological indicators of fecal contamination was determined using SPSS version 15.0 (SPSS Inc., Chicago, IL, USA). The values of fecal coliform counts and number of *E. coli* were converted to logarithmic forms, and the correlation coefficient was ­estimated. Association was analyzed using Pearson’s Chi-squared test. Results with p<0.05 were considered as statistically significant.

## Results

### Prevalence of EV in seafood

**Figure-1:**
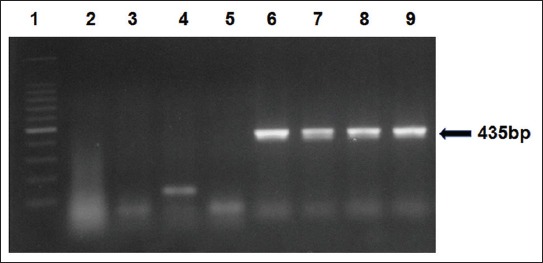
Detection of enterovirus (EV) RNA by reverse-transcriptase PCR using primers ENV-F and ENV-R1. Lane 1: 100 bp DNA ladder, Lane 2: Negative control, Lanes 3-5: EV-negative samples, Lane 6: *Meretrix meretrix*, Lane 7: *Perna viridis*, Lane 8: *Villorita cyprinoides*, Lane 9: Positive control.

**Figure-2:**
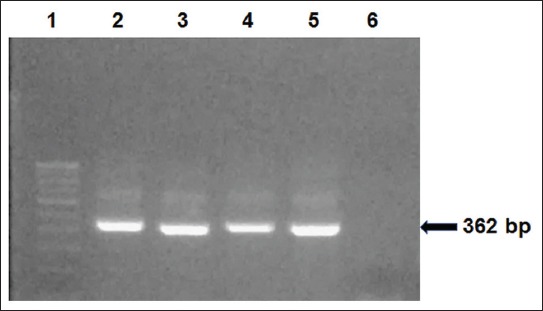
Amplification of enterovirus nucleic acid using nested primers ENV-F and ENV-R2. Lane 1: 100 bp DNA ladder, Lane 2: Positive control, Lanes 3 and 4: *Saccostrea cucullata*, Lane 5: *Meretrix meretrix*, Lane 6: Negative control.

The EV-specific first step primers ENV-F and ENV-R1 yielded a 435 bp amplification product with virus-positive samples (Figure-1). Some samples of shellfish, which were negative in the first step, showed positive amplification of 362 bp with nested primers ENV-F and ENV-R2 (Figure-2). Of 89 seafood samples analyzed by reverse transcription-PCR (RT-PCR), EV RNA was detected in 32 samples with a prevalence of 35.95% (Table-1). Only bivalve shellfish samples were positive for the presence of EV by PCR, while none of the crustacean shellfish and finfish samples was positive for the presence of these viruses (Table-1). The prevalence was found to be highest in black clams with 48.4% of samples being positive for EV. Of 16 samples of oysters analyzed, EV was detected in 9 samples, while 6 of the 19 samples of Asiatic hard clams tested positive for the viral RNA (Table-1). Enterovirus RNA was also detected in one sample each of green mussel and marsh clam (Table-1). Among the samples tested, enteroviruses were predominantly detected in samples collected from open and retail markets of Mumbai.

**Table-1:** Occurrence of EV in different samples analyzed in this study.

Sample	Scientific name	Number of samples analyzed	Number positive for EV*
Bivalves			
Black clam	*Villorita cyprinoides*	31	15
Rock oyster	*Saccostrea cucullata*	10	7
Cupped oyster	*Crassostrea gryphoides*	6	2
Asiatic hard clam	*Meretrix meretrix*	19	6
Green mussel	*Perna viridis*	2	1
Marsh clam	*Polymesoda erosa*	1	1
Crustaceans			
Indian white prawn	*Penaeus indicus*	2	0
Speckled shrimp	*Metapenaeus monoceros*	8	0
Paste shrimp	*Acetes* spp.	3	0
Finfish			
Indian anchovy	*Stolephorus indicus*	2	0
Oil sardine	*Sardinella longiceps*	3	0
Indian Mackerel	*Rastrelliger kanagurta*	2	0
Total		89	32 (35.95)

* Based on the detection of viral nucleic acid. EV=Enterovirus

### Relationship between EV detection and fecal indicator bacteria

All (100%) the samples analyzed in this study were positive for fecal coliforms by MPN method (Table-2). The samples positive for enteroviruses were analyzed further for correlation with the fecal coliform load (Table-2). Among the samples positive for EV, 52.17% of the samples harbored fecal coliforms ranging in counts from 20 to 100 MPN/100 g of the sample, while 37.84% and 8% of the samples harbored fecal coliforms counts of <20 and >100 MPN/100 g, respectively (Table-2). However, majority of the samples showed *E. coli* counts of <4 MPN/100 g of the sample, while 25.92% and 16.6% of the samples showed *E. coli* loads of 4-8 and >8 MPN per 100 g of the sample, respectively (Table-2).

**Table-2:** Relationship between fecal coliform load and the incidence of enteric viruses in seafood samples.

Fecal coliform count	Number of samples tested	Number (%) positive for EV*	r value (correlation coefficient)
<20	37	14 (37.8)	0.839
20-100	23	12 (52.1)	
>100	25	2 (8)	
*E. coli* count			
<4	22	15 (68.1)	0.813
4-8	27	7 (25.9)	
>8	36	6 (16.6)	

* Based on the detection of viral nucleic acid. EV=Enterovirus, *E. coli=Escherichia coli*

Statistical analysis revealed a significant positive correlation (0.839) between fecal coliforms and the presence of enterovirus in samples. Correlation between *E. coli* isolation and detection of enterovirus was found to be positive and significant (0.813) (Table-2).

## Discussion

Enteric viruses are an important group of organisms responsible for causing gastrointestinal infections in humans. Several outbreaks of enteric viral infections linked to contaminated shellfish, especially bivalves, have been reported globally [18,19]. The present study was aimed to determine the prevalence of EV in seafood from Mumbai, India. The samples included seafood intended for human consumption such as shellfish (oysters, clams, mussels, and shrimps) and finfish (sardine, mackerel, anchovy etc.). The coastal water off Mumbai, especially the creeks, is prone to fecal contamination through the discharge of sewage and anthropogenic activities by human settlement close to the coastal water. Shellfish harboring EV were either directly harvested from such contaminated waters or might have got contaminated with EV during various stages of handling and ­transportation. The results of this study also hold significance in view of the very limited studies on the occurrence of enteric viruses in seafood in India. Umesha *et al*. [9] reported the occurrence of EVs in 37% of oyster, 46% of clam, and 15% of shrimp samples and adenovirus in 17% of oyster and 27% of clam samples collected from the south-west coast of India. In our study, 48.4% of the clam samples were positive for EVs, with an overall prevalence of 35.9% of EVs among all the samples screened. Le Guyader *et al*. [20] reported that 21% of oyster samples and 45% of mussel samples were contaminated with EVs along with other enteric viruses during a 3-year study. In a study by Mesquita *et al*. [21] in Portugal, EVs were detected in 35% of the shellfish analyzed. EVs were detected in 43.9% of bivalve mollusks screened from the northwestern coast of Spain [22].

The high prevalence of EV in bivalves such as clams and oysters can be attributed to their filter feeding habit which enables them to accumulate enteric viruses from the surrounding water [13]. The presence of enteric viruses in bivalves collected from fish landing centers and retail supermarkets indicates that they are marketed without adequate depuration process to remove these pathogens. Although sewage treatment processes have improved, still large volumes of sewage enter into the coastal waters leading to the contamination of shellfish in the region of this study. Individuals who consume such contaminated shellfish are at risk of gastrointestinal infections. Several reports have been published describing the outbreak of acute gastroenteritis from enteric viruses in humans related to consumption of seafood, especially bivalve shellfish globally [23]. Foodborne gastroenteritis outbreak due to enteric viruses is substantially underreported in India due to inadequate facilities for source tracking and lack of research efforts in this field.

Currently, fecal coliforms and *E. coli* are used as indicators of fecal contamination of food and water. Indicator bacteria are very useful for monitoring the contamination status of coastal water and the seafood. However, studies suggest that fecal indicator bacteria are not reliable indicators of the presence of enteric viruses [9,24]. The low levels of correlation between fecal indicators and enteric viruses have further consolidated this hypothesis. The reason for low correlation has been attributed to slow depuration rate of enteric viruses compared to the pathogenic bacteria. This may be true when the level of fecal contamination is very low, which provides ample scope for natural depuration. We observed a positive correlation between fecal indicator bacteria and the presence of EVs (Table-2). The correlation values strongly suggest an association between the presence of enteroviruses and the bacteriological quality evaluated. This observation is in accordance with Moreno *et al*. [23], although it is contradictory to the observations of few others [9,21]. One possible factor affecting the high correlation could be different microbial densities in the original contamination sources [24]. The presence of enteroviruses in the samples studied strongly suggests high levels of fecal pollution in the area of sampling in this study. The high correlation between EV, fecal coliforms, and *E. coli* may be attributed to this persistent and perhaps, recent fecal contamination of seafood. However, the use of fecal coliforms or *E. coli* as indicators of EV in shellfish needs to be cautiously analyzed. Our results suggest that 52.17% of the samples positive for EV harbored median level (20-100 MPN/100 g) fecal coliform counts (Table-2), while 68.18% of the samples with *E. coli* counts of <4 MPN/100 g were positive for EV (Table-2). A study on coastal seawater samples from Santa Monica Bay, CA over a 6-year period showed no significant correlation between the presence of EVs and total coliforms, fecal coliforms, or enterococci [25]. It may be worthwhile to explore alternate indicators of EV presence in seafood and coastal waters. Recent studies have shown the utility of human-specific coliphages as good indicators of human viral pathogens [26].

## Conclusion

Our study suggests the contamination of seafood and shellfish in particular, harvested and sold off Mumbai coast with EVs. Virus concentration followed by RT-PCR could be successfully used to detect the presence of EVs in seafood. Cell culture assays take weeks to months to perform and are not suitable for regular monitoring of food and water samples [27]. Moreover, there is no single cell line that allows the propagation of diverse EV species. RT-PCR assay is widely followed for the detection of enteric viruses in fish and shellfish samples and is considered reliable for routine monitoring of samples. Further studies are necessary to determine the prevalence of different groups of enteric viruses in seafood and the critical points of contamination and to establish an effective indicator of viral contamination for tropical seafood.

## Authors’ Contributions

BBN conceived and designed the experiments; ML performed the experiments. OD assisted in sampling and molecular analysis; BBN, SK, and ML planned the experiments and analyzed the data. All authors read and approved the final manuscript.
